# Stigma among tuberculosis patients and associated factors in urban slum populations in Uganda

**DOI:** 10.4314/ahs.v21i4.18

**Published:** 2021-12

**Authors:** Clare Ashaba, David Musoke, Solomon Tsebeni Wafula, Joseph Konde-Lule

**Affiliations:** 1 Department of Health Policy, Planning and Management, School of Public Health, Makerere University College of Health Sciences, Kampala, Uganda; 2 Department of Disease Control and Environmental Health, School of Public Health, Makerere University College of Health Sciences, Kampala, Uganda; 3 Department of Epidemiology and Biostatistics, School of Public Health, Makerere University College of Health Sciences, Kampala, Uganda

**Keywords:** Stigma, tuberculosis, health facility, urban population, Uganda

## Abstract

**Background:**

Stigma continues to be a major barrier to tuberculosis (TB) control particularly in urban populations. Stigma can influence health seeking behaviour and affect adherence to TB treatment, yet few studies have examined TB related stigma and associated factors in Uganda. This study was therefore conducted to determine the level of stigma and associated factors among TB patients in an urban setting in Kampala, Uganda.

**Methods:**

A cross-sectional study was conducted in Makindye division, Kampala among 204 patients with TB aged 18 years and above. Data were collected on socio-demographic, individual patient and HIV/AIDS related factors using an interviewer administered questionnaire. The outcome variable (stigma) was assessed on a four-point Likert scale from the participants' perspective. Stigma scores ranged from 0 to 36 which were summed up and a median stigma score calculated. Individuals with a stigma score equal or greater than the median were categorized as having high stigma. A multivariable logistic regression analysis was performed to determine factors associated with TB stigma.

**Results:**

Over half (52%) of the participants were found to have high TB stigma. Knowing someone who had died of TBAOR = 4.42, 95% CI (1.69 – 11.50) and believing that TB and HIV symptoms were similarAOR = 3.05, 95% CI (1.29 – 7.22) were positively associated with high TB stigma. The odds of having high stigma were 79% lower among individuals who had been previously treated for TBAOR = 0.21, 95% CI (0.09 – 0.52).

**Conclusions:**

Stigma towards TB was high in this urban population and mainly associated with knowing a person who had died of TB, perception that symptoms of TB are similar to those of HIV/AIDS, and previous TB treatment. Interventions to mitigate TB stigma are needed in urban populations and should also address HIV/AIDS related stigma.

## Background

### Burden of TB

alth threat, and ending the epidemic by 2030 is among the health targets of the sustainable development goals^2^. In 2018 an estimated 10 million people fell ill with TB worldwideover 1.5 million patients died from the disease with an estimated 58 million ives saved through timely diagnosis and treatment between 2000 and 2018. Although TB incidence is declining globally, there is need to accelerate this decline in order to reach the 2020 milestones of the End TB Strategy. It should be noted that people living with HIV are 19 times more likely to develop active TB disease than people without HIV^2^. The 2017 global tuberculosis report places Uganda among the 30 countries with the highest burden of TB / HIV co-infections. Moreover, approximately 86,000 cases were registered, and 11,000 people died of TB in Uganda^2^. Worse still, TB management in Uganda is challenged by low rates of case detection at 52% and of treatment success at 75%^2^.

### TB Stigma

Although treatment of TB is affected by various biological, cultural and economic factors, stigma continues to be a major social factor affecting compliance to treatment among patients, and influencing their health seeking behaviours^4^–^6^. Individuals who are living with TB should be central to the TB response. Understanding their personal experiences will create momentum for better policies, drugs and diagnostics as well as accelerate investment for the disease^51^, ^52^. Stigma was originally defined by Goffman (1963) as “an undesirable or discrediting attribute that an individual possesses thus reducing that individual's status in the eyes of society”7. More recently, health related stigma has been defined as “a social process or related personal experience characterized by exclusion, rejection, blame, or devaluation that results from experience or reasonable anticipation of an adverse social judgment about a person or group identified with a particular health problem”8. This definition highlights two components of stigma: patient experiences of discrimination in their communities; and the fear and anticipation of social consequences with or without the actual experience by the patient. Therefore, defining stigma as a social process highlights the importance of interactions between those who have the health problem (the patient) and those who do not (the healthy community members) 9.

TB is viewed as a stigmatizing disease because of its associations with marginalized groups such as the poor^10^, ^11^, ^51^, ethnic minorities^12^, low social class^13^, ^14^, prisoners and refugees^11^, ^15^, and HIV/AIDS patients^12^,^16^, ^17^. The impact of stigma can be felt at home, in the workplace, at health facilities, and in the community^13^, ^18^. Patients with TB report concerns about discrimination, isolation and rejection. In additon, patients report fears of divorce, little or no marriage prospects, not being able to share meals and utensils with other household members, and being the subjects of gossip in the community^19^,^20^. Such fears may lead to delays in seeking care for TB symptoms, and may affect adherence to treatment. It is as a result of the above factors that TB stigma is viewed as a barrier to TB control^21^. Previous studies indicate that TB stigma may be influenced by limited knowledge about TB cause, transmission, diagnosis and treatment^22^–^25^. In addition, women have been found to experience the effects of stigma more often than men^13^,^26^, ^27^. Stigma towards TB has also been shown to be a significant barrier to TB control in Uganda^21^, ^28^. This study was therefore undertaken to assess the level of TB stigma and associated factors among TB patients in Makindye division, Kampala, Uganda.

## Methods

### Study area and context

The study was carried out in Makindye division, one of the five divisions of Kampala, Uganda's capital city. Makindye is located in the south-eastern region of Kampala and comprises 21 parishes with a population of 439,300 people29. This study was conducted at four health facilities in the division, including: Kisugu Health Center, Touch Namuwongo Clinic, Alive Medical Center, and Nsambya Homecare Clinic. These health facilities were purposively selected as they had the highest patient volumeCA3 in the division. Kisugu Health Center is operated by Kampala Capital City Authority (KCCA) whereas Alive Medical Centre, Touch Namuwongo and Nsambya Homecare Clinic are private-not-for-profit facilities. All these facilities offer outpatient services which include smear microscopy and TB treatment as well as have integrated HIV and TB services. Patients seen at these clinics are typically walk-ins and referrals from practitioners within and outside Kampala.

### Study design, population and sample siz

The study design was cross sectional using quantitative methods and was conducted between April and June 2014.CA4 The study sample was drawn from all the TB patients that were registered for treatment at the four health facilities and included only patients aged 18 years and above. Initial sample size of 382 was obtained using the formula by Kish Leslie with Zα = 1.96, p = 0.47 from a previous study30 and a precision (σ) of 5%. Since the total number of TB patients that were receiving treatment in health facilities in the division was approximately equal to the estimated sample size, the computed sample size was adjusted since it was not possible to include all the TB patients in the study due to decline to participate and other reasons. A formula for sample size calculation was therefore used to obtain a new sample size (SS): new SS = SS / (1+ (SS-1)/population size of TB patients. Therefore, new SS = 382 /(1+ (382-1)/400) = 196. Considering, a non-response rate of 10%, a final sample size of 217 participants was obtained.

### Data collection and sampling

A semi-structured questionnaire was designed based on reviewed literature^13^, ^31^–^33^. It included information on socio-demographic characteristics such as sex, age, marital status, level of education as well as questions on individual factors including socio-demographics, TB related knowledge, practices and beliefs, previous history of TB as well as HIV/AIDS related factors associated with TB stigma. Research assistants with prior experience in conducting facility based investigations were .trained on appropriate techniques of data collection, and supervised to ensure compliance with the study protocol. The study tools were pretested in a health facility in the neighbouring division, and later refined before actual data collection. All TB patients who met the inclusion criteria were consecutively enrolled into the study until the sample size was realized. Patients who withdrew consent or were too ill to consent were excluded from the study.

### Data entry and statistical analysis

Data were entered in Epi data version 3.1 (EpiData Association, Denmark), and later exported to Stata version 12 (StataCorp, Texas, USA) for cleaning and analysis. Descriptive statistics were used to run frequencies and proportions of socio-demographic characteristics, knowledge on TB and experiences of TB stigma. The dependent variable for this study was TB stigma. The scale used to measure stigma was adopted from reviewed literature^31^. This scale demonstrated a high internal consistency, and has been used to measure stigma in populations similar to our study^32^. The scale measured the perceived (internalized / felt) stigma among patients with TB. Perceived stigma measures the experiences, thoughts and feelings of patients with TB. Stigma items were scored on a Likert scale with four levels: strongly disagree (0), disagree (1), agree (2), and strongly agree (3). Higher scores indicated a greater degree of stigma towards TB. Responses were summed to create a stigma score for each respondent, and a/span> median stigma score was calculated. Individuals with a stigma score equal or greater than the median score were categorized as having high stigma while those with a stigma score below the median were categorized as having low stigma towards TB. Logistic regression was used to determine the factors associated with TB stigma. Bivariate analysis was used to obtain the crude odds ratios (COR) for association between the independent variables and the outcome (TB stigma) at 95% confidence interval. The independent variables that were significant with p<0.05, those that had marginal associations (p<0.20) in the bivariable analysis, and factors known to influence TB stigma from literature were considered in forward stepwise logistic regression. The adjusted odds ratios (AOR), their confidence intervals and p values in the final model have been presented.

## Results

### Socio-demographic characteristics of study participants

A total of 204CA9 patients participated in the survey representing a 94.0% response rate. More than half 56.9% (116/204) were male and Catholics 51.5% (105/204). Nearly forty percent 39.7% (81/204) of the participants were never married, and 33.2% (66/204) unemployed. The median age of participants was 30 years and ranged from 18 to 76 years (Table 1).

**Table 1:** Socio-demographic characteristics of study participants

Socio-demographic factors	Frequency (N = 204)	Percentage (%)
**Sex**		
Male	116	56.9
Female	88	43.1
**Age (median, range)**	30, (18 – 76)	
**Marital status**		
Never married	81	39.7
Currently married / living together	77	37.8
Formerly married / widowed	46	22.5
**Level of education**		
None	10	4.9
Primary	69	33.8
Secondary	81	39.7
Tertiary	44	21.6
**Occupation**		
Unemployed	66	32.3
Paid employment	52	25.5
Self-employed	86	42.2
**Religion**		
Muslim	32	15.7
Catholic	105	51.5
Protestant	50	24.5
Other *	17	8.3

* Other included Pentecostals and Seventh Day Adventists

### Knowledge of tuberculosis transmission and HIV/AIDS status

The majority of participants knew that TB was spread by infection from others 94.6% (193/204), could be transmitted through coughing and sneezing 98.5% (201/204), and that TB was curable 97.1% (198/204). Most participants thought that TB could be transmitted by: eating and drinking with infected persons 77.5% (158/204); smoking and drinking alcohol 78.4% (160/204); sexual intercourse 66.2% (138/204); and touching an infected person 68.6% (140/204). Less than half 47.6% (97/204) of the participants had been previously treated for TB. In addition, majority of participants had tested for HIV/AIDS 95.1% (194/204) with nearly half 49.0% (95/194) of them being HIV positive (Table 2).

**Table 2:** Knowledge on tuberculosis transmission and HIV/AIDS status

Variables	Frequency (N = 204)	Percentage (%)
**Knowledge on TB cause / transmission:** **A person can get TB infection from others**		
Yes	193	94.6
No	05	2.5
Did not know	06	2.9
**A person can get TB through eating and drinking with others**		
No	20	9.8
Yes	158	77.4
Did not know	26	12.8
**One can get TB through smoking and/or drinking alcohol**		
No	23	11.3
Yes	160	78.4
Did not know	21	10.3
**TB can be hereditary**		
No	65	31.9
Yes	62	30.4
Did not know	77	37.8
**TB can be transmitted through inhaling cough and sneeze** **aerosols**		
Yes	201	98.5
No	03	1.5
**TB can be spread through sexual intercourse**		
No	39	19.1
Yes	135	66.2
Did not know	30	14.7
**TB infection through touching others**		
No	140	68.6
Yes	40	19.6
Did not know	24	11.8
TB Knowledge (Cure)		
**A person with TB can be cured**		
Yes	198	97.1
No	2	1.0
Did not know	4	1.9
**Previous treatment for TB**		
No	107	52.4
Yes	97	47.6
**Directly Observed Treatment (DOTS)**		
Yes	111	54.4
No	93	45.6
**Tested for HIV**		
Yes	194	95.1
No	10	4.9
**HIV status (n = 194)**		
Negative	99	51.0
Positive	95	49.0

### Level of TB stigma and associated factors

The median TB stigma score was 22 and ranged from 3 to 36. Over half 106/204 (52.0%) of the participants had high stigma. Bivariable analysis results indicated that participants who were married or cohabiting COR= 2.97, 95% CI (1.55 – 5.68), formerly married or widowed COR = 5.08, 95% CI (2.30 – 11.19), and Catholics by religionCOR = 3.17, 95% CI (1.37 – 7.37) were more likely to have high TB stigma. Regarding knowledge, we found that participants who thought that TB could be transmitted through smoking and drinking alcoholCOR = 4.36, 95% CI (1.63 – 11.67), TB transmission was hereditaryCOR = 2.86, 95% CI (1.34 – 6.09), or transmitted sexuallyCOR = 2.72, 95% CI (1.31 – 5.66) were more likely to have TB stigma. Individual and HIV/AIDS risk factors for TB stigma included knowing someone who died of TBCOR = 5.13, 95% CI (2.66 – 9.89), being HIV/AIDS positiveCOR = 2.05, 95% CI (1.15 – 3.64) and thinking that TB and HIV symptoms appear similarCOR = 4.22, 95% CI (2.27 – 7.84) which were all associated with TB stigma. Participants with previous TB treatment were less likely to have high TB stigmaCOR = 0.18, 95% CI (0.10 – 0.33). After controlling for potential confounders, the final multivariable logistic regression model indicated that the odds of having high TB stigma were four times higher among individuals who knew someone who died of TB compared to those who did notAOR = 4.42, 95% CI (1.69 - 11.50). Participants who had previously been treated for TB were 79% less likely to have high TB stigma compared to non-rereatment casesAOR = 0.21, 95% CI (0.09 – 0.52). However, participants who believed that TB and HIV symptoms appear similar were three times more likely to suffer with high TB stigma compared to those who did not think soAOR = 3.05, 95% CI (1.29 – 7.22) (Table 3).

**Table 3:** Independent predictors of high stigma among TB patients

Variables	Number of participants with high TB stigma (%)	AOR (95% CI)	p-value
**Marital status**			
Never married	27 (33.3)	1	
Married / living together	46 (59.7)	1.05 (0.41–2.66)	0.925
Formerly married / widowed	33 (71.7)	2.86 (0.92–8.91)	0.071
**Religion**			
Muslim	10 (31.3)	1	
Catholic	62 (59.0)	2.46 (0.76–7.99)	0.134
Protestant	25 (50.0)	1.92 (0.47–7.92)	0.364
Other	09 (52.9)	1.62 (0.28–9.25)	0.585
**TB knowledge (transmission):** **Eating / drinking with others**			
No	8 (40.0)	1	
Yes	93 (58.9)	0.49 (0.11–2.33)	0.377
Did not know	05 (19.2)	0.49 (0.07–3.39)	0.470
**TB spread through smoking /** **alcohol**			
No	6 (26.1)	1	
Yes	97 (60.6)	2.74 (0.54–14.04)	0.226
Did not know	3 (14.3)	0.89 (0.11–7.59)	0.922
**TB spread through sexual** **intercourse**			
No	15 (38.5)	1	
Yes	85 (63.0)	2.46 (0.85–7.13)	0.098
Did not know	6 (20.0)	0.79 (0.18–3.59)	0.770
**Knew someone who died of TB**			
No	53 (39.3)	1	
Yes	53 (76.8)	4.42 (1.69–11.50)	0.002*
**Previous TB treatment**			
No	76 (71.0)	1	
Yes	30 (30.9)	0.21 (0.09–0.52)	0.001*
**Directly Observed Treatment** **(DOTs)**			
Yes	52 (46.8)	1	
No	54 (58.1)	1.22 (0.52–2.84)	0.651
**HIV status**			
Negative	44 (44.4)	1	
Positive	28 (62.1)	1.17 (0.47–2.91)	0.739
**TB and HIV appear similar**			
No	21 (29.6)	1	
Yes	85 (63.9)	3.05 (1.29–7.22)	0.011*

* Significant findings p-value <0.05

## Discussion

The study assessed the level of TB stigma and associated factors among TB patients in an urban population in UgandaCA10. Our study showed that over half of the participants in this urban setting had high stigma. In the study, knowing someone who died of TB, previous TB treatment, and having a perception that TB and HIV appear similar emerged as predictors for TB stigma. These findings are likely to be important in designing interventions to reduce TB stigma in urban settings in Uganda.

Most participants stated that TB could be spread through drinking alcohol or smoking which is in line with other studies^34^–^36^. One third of the participants stated that TB is hereditary which is also consistent with studies in Philippines^37^ and Indonesia^38^. Associating TB with alcohol consumption, smoking and heredity is a misconception as Mycobacterium tuberculosis, the bacteria that causes the disease, is spread through air when an infected person releases the organisms for example through coughing. Furthermore, our study found that most participants thought that having sexual intercourse with a TB infected person could result into TB infection corroborating with findings from a study in Zambia^39^. Although our study did not assess the link between TB and sexual behaviour, a survey in the United States of America found many HIV negative TB patients reporting risky sexual behaviours^40^ which warrants further investigation. Despite the above misconceptions about the causes and transmission of TB, many participants also knew that TB could be transmitted from one person to another, and that it could be cured. These gaps in knowledge underscore the need for more robust health education programmes to stress the correct cause and transmission of TB among patients and the general population.

Our study found that more than half of the participants suffered high TB stigma. This finding is consistent with those of other studies in Ethiopia 32 and Uganda^30^ which also reported high TB stigma levels. The levels of TB stigma observed in our study are however higher than those reported in another study in Sudan^41^. The low level of TB stigma in the Sudan study has been attributed to the generally unique social-cultural nature of the people who reportedly maintain close social ties, even more strongly during times of illnesses^41^. High TB stigma in several countries emphasises the need to address this public health problem especially in high burden countries including Uganda.

Nearly half of the TB patients in our study were co-infected with HIVCA^11^. This proportion is higher than what has been found by other studies^42^–^44^. The higher rate of TB-HIV co-infection in our study is likely to be due to the lower prevalence of HIV in the countries where the other studies were carried out including India and China. Having both TB and HIV has been shown to have profound effects on the body's immune system since they are capable of disarming the host's immune responses^45^. This finding of TB-HIV co-infection therefore indicates the need to promote integration of HIV/TB programmes to improve health outcomes among patients of both diseases.

Regarding predictors of TB stigma, individuals who knew someone who died of TB were significantly more likely to have high TB stigma. This relationship may partly be explained by the fact that whereas TB deaths may be independent, a section of the public associates them with those of HIV/AIDS. Indeed, a study on TBHIV stigma in high HIV prevalence settings in Zambia confirmed that TB stigma may be compounded by HIV stigma^19^. Moreover, in areas of high HIV prevalence, it has been suggested that a TB diagnosis could have been used as an attempt to hide one's HIV status by health workers, the patients and family members during the course of illness and at funerals^19^. These findings are consistent with those of a similar study which found that knowing someone who died of TB had the strongest and most precise association with higher TB stigma^46^. This finding therefore suggests that interventions aimed at reducing TB stigma will impact positively on the uptake of TB/HIV services.

In our study, the belief among participants that TB and HIV symptoms appear similar was significantly associated with higher TB stigma. This finding further highlights the effect of HIV and HIV stigma on pre-existing TB stigma. In the context of HIV, TB symptoms and diagnosis are often accompanied by the assumption that HIV co-infection is very likely. In addition, progressive, severe and reoccurring TB usually confirms community's perceived diagnosis of HIV. Indeed, symptoms like extreme weight loss, productive cough and some side effects of TB treatment such as skin rash further confound the perception that a TB patient is HIV positive^19^. Most prior studies on TB stigma have been qualitative and have highlighted the fact that TB is stigmatized due to its associations with HIV/AIDS^26^, ^47^. Indeed, a qualitative study in Thailand identified symptoms similar to HIV/AIDS as one of the reasons for stigmatizing TB^25^. Findings from this study are similar to our results and consistent with other studies^30^, 48. Our findings further highlight the importance of integration of TB and HIV programmes, and the issues that could be addressed to reduce TB stigma.

Previous treatment for TB was negatively associated with high TB stigma as retreatment cases were significantly less likely to have high TB stigma. It is widely believed that TB stigma negatively affects adherence to treatment^47^. The protective nature of previous treatment may be partly explained by the possibility that TB patients that had been previously treated and interacted with other TB patients were knowledgeable about the disease^49^ and had experience in managing it and its associated stigma. Future studies could investigate whether retreatment patients may seek treatment far from their homes which could also reduce stigma. Retreatment cases also have a higher likelihood of developing multi-drug resistant (MDR) TB due to prior exposure to TB drugs^50^. These findings could be attributed to the strong focus on retreatment cases within TB programmes to improve treatment outcomes and control MDR TB. Additionally, it is possible that due to their prior poor treatment outcomes, the retreatment cases had to a great extent overcome stigma and were hence more compliant in terms of seeking care and adherence to treatment.

This study had limitations that should be noted. This was a facility based study on TB patients enrolled on treatment and care. It is possible that to some extent, these patients had coped with stigma towards the disease. Therefore, low stigma levels in some respondents could be attributed to this. Additionally, higher levels of stigma could have prevented TB patients in the community from seeking care and treatment. It is also important to note that summarizing Likert scores during analysis may have caused a loss of power and inaccurate estimation. This may lead to difficulty comparing results across studies due to the data driven cut points used to define categories. Stigma towards TB has been closely linked to associations of TB to HIV/AIDS yet our study did not measure HIV/AIDS stigma. Therefore, the implications of TB-HIV stigma on TB control may not be fully explored using findings from this study.

## Conclusions

Our study showed that TB stigma was high in this urban population and found to be significantly associated with knowing a person who died of TB, being co-infected with HIV/AIDS and perception that TB symptoms are similar to those of HIV/AIDS. The findings suggest that TB stigma is compounded by HIV stigma which underscores the importance of integrating TB and HIV programmes. More effort is needed to address stigma in TB control programmes.

## Data Availability

The datasets generated and/or analysed during this study are available from the corresponding author on reasonable request.

## Abbreviations

AOR: Adjusted Odds Ratio

COR: Crude Odds Ratio

HDREC: Higher Degrees Research and Ethics Committee

KCCA: Kampala Capital City Authority

LMICs: Low and Middle Income Countries

MDRTB: Multi-drug Resistant Tuberculosis

## Notes

**Declarations**
